# Stigmatizing bioterrorism as a public health concern: The case of anthrax in US media

**DOI:** 10.1186/s12889-026-26851-1

**Published:** 2026-03-06

**Authors:** Juan Martin Dabezies, Nickolas Freeman, Natalia Muñoz Cassolis, Judith J. Rakowski, Elle Jingjing Xu, Meredith L. Gore

**Affiliations:** 1 Department of Geographical Sciences, University of Maryland College Park, College Park, MD USA; 2 Department of Agrarian Systems and Cultural Landscapes, University of the Republic, Rocha, Uruguay; 3 Department of Information Systems, Statistics, and Management Science, University of Alabama, Tuscaloosa, USA

**Keywords:** Communication, Global Health, Natural Language Processing, Communities Detection

## Abstract

This work approaches the role of media in stigmatizing anthrax as a bioweapon in the United States, transitioning the disease from a relatively obscure zoonotic pathogen to a symbol of bioterrorism. By analyzing over 20,000 articles published between 1979 and 2023, we trace how the 2001 anthrax attacks in the US significantly shifted media coverage to focus anthrax as a national security, terrorism, and public health concern. This framing reinforced the stigmatization of anthrax as a militarized threat, even during periods of low assessed risk. The study uses a novel data science approach integrated with classical social science theories to map the evolution of stigmatization themes. The findings highlight how media coverage has come to prioritize framing bioterrorism over public health risks, limiting efforts to manage anthrax as a neglected zoonotic disease. The study underscores an opportunity for more balanced media messaging that reduces stigmatization, minimizes public fear, and promotes awareness of anthrax as both a public health issue and a global health concern.

**Supplementary Information:**

The online version contains supplementary material available at 10.1186/s12889-026-26851-1.

## Introduction

Understanding and managing the role of news media in stigmatizing health issues is of great interest to public health communication [11, 19], and it is broadly evident across various health domains. For example, during the 2014–2016 Ebola outbreak in West Africa, news coverage reported transmission risks while also framing affected communities as “unsafe.” This coupling contributed to fear, discrimination, and social exclusion [22]. The news media’s handling of the HIV/AIDS crisis in the 1980s and 1990s stigmatized the LGBTQ+ community, marginalizing them and complicating public health efforts on transmission prevention [2]. Several studies have analyzed the stigmatization processes at the onset of the COVID-19 pandemic, particularly targeting Asians [21, 25, 41]. These cases help exemplify the extant literature’s focus on studying the relationship between news media and stigmatization, centering on the processes of categorization and marginalization of human groups associated with disease(s). In this work, we enhance the literature by exploring news media stigmatization of a disease itself, in this case, anthrax. Anthrax is a bacterial disease caused by *Bacillus anthracis*, which affects mammals globally, including livestock and wildlife [3]. Global estimates suggest that annually, anthrax causes approximately 20,000–100,000 infections in humans, disproportionately affecting poor rural populations living in close proximity to livestock; an estimated 63.8 million rural poor livestock keepers and over 1 billion livestock live in anthrax-risk areas, underscoring its ongoing public health relevance despite persistent under-recognition and under-reporting [8]. Despite being one of the oldest zoonotic diseases known to health practitioners, anthrax has received relatively little attention from global health organizations compared to other emerging infectious diseases [28]. Yet anthrax has been uniquely stigmatized, as we show below.

In September and October 2001, anthrax was used as a biological weapon in the United States (U.S.) when several anthrax-laden letters were sent via postal mail to media outlets, U.S. senators, and government offices. In total, twenty-two individuals were infected with a weaponized form of anthrax (i.e., in powder form) that was “hidden in plain sight.” Five people died and panic ran high: hoaxes and false alarms involving letters thought to contain *B. anthracis* became common disruptions. The investigative arm of the U.S. Postal Service ultimately dealt with over 17,000 incidents and evacuated over 600 postal facilities in the year following the attacks [10, 12]. These attacks, occurring in the aftermath of the September 11, 2001 terrorist attacks —in which Al-Qaeda hijacked airplanes and crashed them into the Twin Towers in New York City and the Pentagon in Washington, D.C., resulting in thousands of deaths and creating widespread fear— quickly transformed anthrax from a disease that was relatively unknown among the general public in the US into a focal point of public fear and uncertainty. The media’s role in amplifying the fear surrounding anthrax-as-bioterrorism weapon created a situation where bioterrorism, using anthrax in particular, simultaneously became a threat to national security and public health [26]. Several studies have focused on analyzing media coverage of these anthrax attacks [15], considering aspects related to the spread of fear and the generation of anxiety in the American population [38, 39]. Media coverage of the anthrax attacks contributed to the cultural separation of anthrax from other diseases and its framing as a sensitive topic, particularly within the context of bioterrorism [1]. The process of associating anthrax with fear and bioterrorism can also be attributed to its prominent historical role in biological warfare, especially during the Cold War, when anthrax became emblematic of weapons of mass destruction. This combination of historical association, wartime use, and media amplification of risk ultimately framed anthrax –a zoonotic disease– as a symbol of fear and bioterrorism, stigmatizing it as a bioweapon [20]. Despite the ongoing threat of anthrax infections in endemic regions, the stigmatization of anthrax has contributed to a trend among global health initiatives to often prioritize its bioterrorism potential in high-income countries over its management as a neglected zoonotic disease with an ability to impact low and middle-income countries [28]. One outcome of this stigmatization is that global health policy on anthrax has been overwhelmingly targeted away from public health needs in vulnerable communities, where anthrax continues to affect both human and animal health [35].

News media plays a crucial role in reflecting and shaping contemporary social dynamics by highlighting specific issues as sensitive or controversial, and embedding certain topics within broader cultural frameworks that shape what is considered acceptable or controversial [1, 14, 17]. According to Framing Theory [13], the media selects relevant topics and shapes how the public perceives these topics, using words and frames with symbolic meaning. Repeatedly using terms like “threat” or “danger” in relation to certain health activities or behaviors can create associations reinforcing the perception of those issues as morally problematic or deviant from societal norms. This process is further amplified through what Kasperson et al., [24] described as the Social Amplification of Risk Framework, where institutional and media responses can exaggerate public perception of risk, shifting what might initially be a contained issue into a broader social problem. Amplification occurs as certain narratives are repeated, often out of proportion to the actual level of risk, creating an escalating cycle of fear and concern. As the media continues to focus on specific risks, they become part of a larger societal conversation that inflates their significance. Over time, these amplified risks are seen not only as immediate dangers but as ongoing threats that require constant vigilance, thus embedding them deeper into the collective consciousness. Finally, according to Stigma Theory [17], these media-driven associations eventually solidify in the collective imagination, creating a social structure that marks specific topics as unacceptable or dangerous. The stigmatization process involves attaching a set of negative attributes to individuals, behaviors, or topics, leading to their marginalization [23].

Beyond exploring the evolution of anthrax-related news media coverage over time with an eye for stigmatization, this research also aims to help advance understanding of media stigmatization of bioterrorism as a public health concern. Results may have implications for health communication, specifically entry points for designing more effective strategies to balance the dissemination of accurate information while not provoking social anxiety about a specific disease or bioterrorism writ large. Understanding media stigmatization of bioterrorism may also offer new entry points for designing more effective communication strategies, balancing the dissemination of accurate information with managing social anxiety [37]. Designing public policies or measures to control and manage socially sensitive topics can prove cumbersome because stigmatization creates communication challenges from evoked and dynamic emotional responses [31, 32]. Indeed, stigmatization can create major barriers to open dialogue, hinder communication strategies, and complicate structures for effective policymaking [11, 33]. Stigmatization often leads to public debates where moral values are prioritized over scientific evidence. Resultant outcomes of stigmatization include inefficient use of public resources or misguided strategies that fail to address root causes. These strategies may sometimes exacerbate the underlying problems, as with bioterrorism [7].

To these ends, this study seeks to help advance understanding of the mechanisms of stigmatization in the media by exploring how a public health concern evolved into a bioterrorism risk. Building upon classical theories of communication and cultural framing [13, 17, 24], we aim to (a) characterize the representation of stigmatization in anthrax-related articles from 1979 to 2023; and (b) examine how and when stigmatization in media content has evolved. We achieve objectives using a novel data science technique to probe these foundational theories, allowing us to access novel details on stigmatization, tracking the evolution of stigmatization themes in tens of thousands of media sources, signaling emergence and senescence. We analyzed 20,054 press releases from the Nexis Uni™ database, covering the available news media related to anthrax over the past 45 years. We present results for each objective after summarizing the data science approach. We first map the representation of thematic associations in anthrax-related articles over the study period. We then explore the temporal shifts in thematic associations within anthrax-related media coverage.

## Methodology

### Data collection

Data were collected from published news media articles about anthrax on Nexis Uni™, a database offering access to over 15,000 news, business, and legal sources. It supports academic work and critical media studies by providing a deep well of journalistic content from trusted sources. Nexis Uni™ gathers news articles from around the globe. The University of Maryland, College Park McKeldin Library’s catalog enabled access to this database. Database searches were conducted between November 10, 2023, and December 13, 2023. We used two Boolean searches with specific exclusions to avoid false results linked to the American rock band called Anthrax: (1) “Anthrax” and “Bioweapon” and not “band” and not “rock” and not “John Bush” and not “Joey Belladonna,” and (2) “Anthrax” and “Attack” and not “band” and not “rock” and not “John Bush” and not “Joey Belladonna.” The search resulted in 20,054 news media articles on anthrax and attack/bioweapon. We considered articles published between 1979 and 2023. This timeframe corresponds to the oldest data available on Nexis Uni™ and the year in which research was conducted. Search results were filtered for “All available dates,” “All content types,” “Title view,” and “Newspapers.” Search results were not filtered by language, thus our data includes news in all available languages (e.g., English, Dutch, French, German). However, news coverage results for the Boolean searches were predominantly in English, with only a very few exceptions in other languages. Once content had been filtered, it was sorted by “Date (oldest to newest).” All news media articles identified through the search were downloaded in Rich Text Format and saved as individual files. Each file included information for a single article, including the title, the source, the full text of the document, the number of times the search keywords were present in the text, and a set of subjects assigned by Nexis Uni™ platform for categorization. A download control table was kept to avoid duplication of articles, and our data analysis code explicitly checked for and dropped any duplicate records.

### Data analysis

We followed an iterative analytic strategy, using sensitizing concepts from the literature while allowing inductive patterns in the corpus to shape and refine our interpretations. To operationalize this strategy, we conducted two complementary analyses of the 20,054 news media articles. The first analysis considered subject classifications assigned to articles by Nexis Uni™ to identify clusters, or communities, of subjects likely to co-occur, indicating they tend to be related in the media discourse. For this analysis, we used the Leiden Community Detection Method. We then used techniques from Natural Language Processing (NLP), such as Term Frequency-Inverse Document Frequency (TF-IDF), and ChatGPT with human assistance, to investigate the text of articles to understand dominant themes and how they have changed over time. Both analyses exhibit different levels of granularity, as the first is based on subjects pre-assigned by Nexis Uni™, while the second focuses on the textual content of the news articles. These analyses are complementary for studying processes of stigmatization, as one examines the characteristics of affinities between sets of topics, defining thematic communities and analyzing their relationships, while the other seeks to understand the functioning of thematic associations in the content over time.

#### Exploring subject communities across the study period

To achieve our first objective, we analyzed broad trends in subject classifications (i.e., topics). ‘Subjects’ in Nexis Uni™ are thematic tags assigned by the platform to help categorize and organize news media articles. Subject tags represent the main topic(s) covered in the content, such as politics, economics, technology, or environmental issues. For each subject assigned to an article, Nexis Uni™ also assigned a relevance score, expressed as a percentage representing the strength of the association between the assigned subject and the article content. We extracted the subject(s) that achieved the highest relevance score for each article. The decision to limit our analysis to each article’s most relevant subject(s) was driven by two key considerations. First, Nexis Uni™ often assigns multiple subjects to a single article, many of which have low relevance scores. By keeping only the most relevant subjects, we aimed to retain those tags that best captured the core themes of each piece. Second, the exact methodology Nexis Uni™ uses to determine relevance scores is not publicly available, making it difficult to establish a precise threshold for selecting subjects (e.g., only those with relevance above 70%). However, it is reasonable to assume that subjects with the highest relevance scores were the most important to the content of each article.

**Fig. 1:**
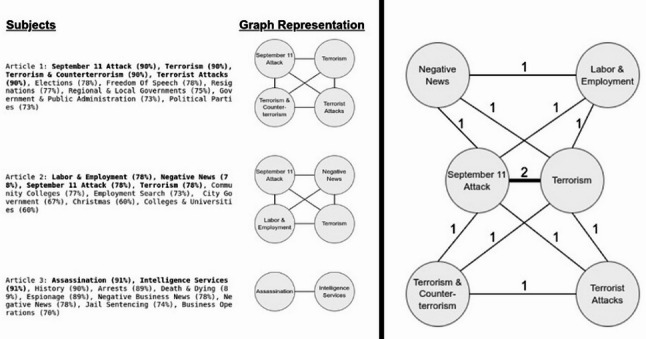
*Left*: We first constructed individual graphs of nodes and edges using platform-identified subjects for individual news media articles from the sample of 20,054; the three example graphs from articles to the right illustrate the diversity of news media article subjects. *Right*: We then constructed an aggregate graph combining all the nodes and edges for sample news media articles; here, an example graph aggregating the three articles illustrates the inclusion of edge weights between most pairs. The edge weights of most vertices are one, indicating that we observed the pair of subjects occurring as a top subject for the same article once. The weight of the edge connecting the subjects “September 11 Attack” and “Terrorism” is two because we observed this pair of subjects occurring as top subjects in articles 1 and 2

We used the subject(s) associated with the highest percentage score for each article to construct a graphical representation of subject relationships. In the graphical representation (Fig. 1), vertices correspond to subjects observed across the total sample of news media articles, and undirected edges connect pairs of subjects that co-occur as top subjects in the same articles. Since we had the opportunity to observe subject pairs co-occurring as top subjects in more than one article, we weighted the edges of the graph based on the frequency with which we observed the co-occurrence for each pair of connected subjects. Higher edge weights represent subject pairs that co-occur more frequently in the sample (Fig. 1).

We apply the Leiden Community Detection Method [40] (also known as Leiden method), to the aggregated graph representation of all news media article subjects to identify groups of related subjects based on their connectivity within the graph. In network science, community detection methods cluster vertices by finding subgraphs with high internal connectivity, as determined by an objective function. The Leiden method ensures identified communities are well-connected and is very computationally efficient, making it suitable for large networks. By optimizing a metric known as modularity, the Leiden method effectively identifies significant relationships between subjects and groups them into communities.

#### Variation in thematic content over time

We achieved our second objective using an analysis developed via blocks of text from the documents within each of the 45 years of the study period. Unlike the analysis used to achieve objective 1, in this case, we did not work with platform-identified subjects but directly with the content and using a chronological perspective. This analysis was based on a combination of the TF-IDF method [34] with other NLP techniques. Specifically, TF-IDF is used to calculate the importance of words or phrases within a document relative to their frequency across the collection of documents. Based on TF-IDF, we created a historical dataset (Supplementary Table 1), which served as the basis for the NLP processing. The specific NLP analysis was done using ChatGPT4.0 with human assistance. The NLP approach analyzes semantic similarity, comparing terms based on their meanings to identify connections and groupings. This involved assessing the context in which each word or phrase appeared, considering nuances in language that could influence their classification. The thematic categories were then constructed by grouping related terms based on this semantic similarity and contextual association, ensuring that terms with shared meanings or related topics were clustered. Once the thematic blocks were defined, temporal annotation was applied to link each category to specific year(s) in which those terms were relevant. Finally, the information was organized into a structured data table using a hierarchical approach, clearly presenting the relationships between thematic categories, their associated terms, and relevant years. This approach combines qualitative analysis, semantic clustering through NLP techniques, temporal annotation, and data structuring to map the evolution of these themes over time.

## Results

### Subject communities across the study period

**Fig. 2:**
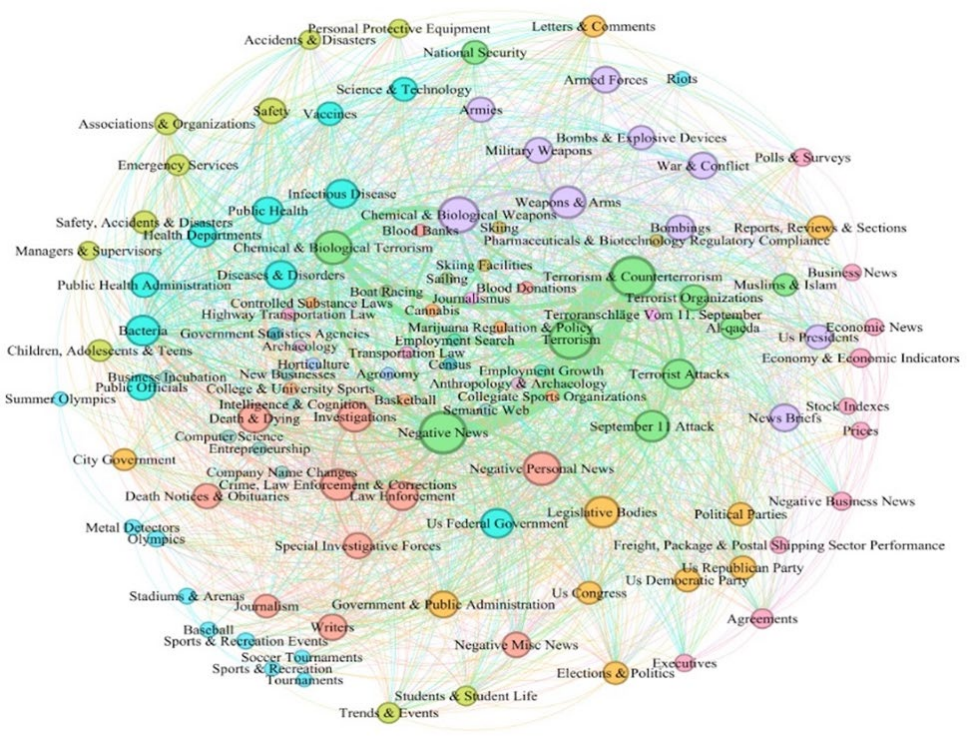
A graphical representation of the 20 communities identified amongst the 20,054 anthrax-related news media articles published over the past 45 years. Vertex color indicates community membership, with vertices of the same color belonging to the same community. The thickness of the edges connecting vertex pairs is proportional to the edge weights, representing the strength of connections between subject pairs. The size of each vertex corresponds to the associated subject’s influence, which we estimated using eigenvector centrality

The Leiden method identified 20 distinct subject communities amongst the 20,054 news media articles published over 45 years. Community detection results for a subset of the graph (Fig. 2) include the vertices and edges corresponding to each community’s ten most important subjects. We chose to show the ten most important subjects to highlight the diversity of subjects in the communities while ensuring the visualization remains readable. To measure the influence of vertices, we chose Eigenvector Centrality Analysis, which measures the influence of vertices based on node connections or edges. Eigenvector centrality is computed iteratively, and connections to other vertices with high eigenvector centrality scores contribute more to the score of a target vertex than connections to low-scoring vertices [5].

**Fig. 3:**
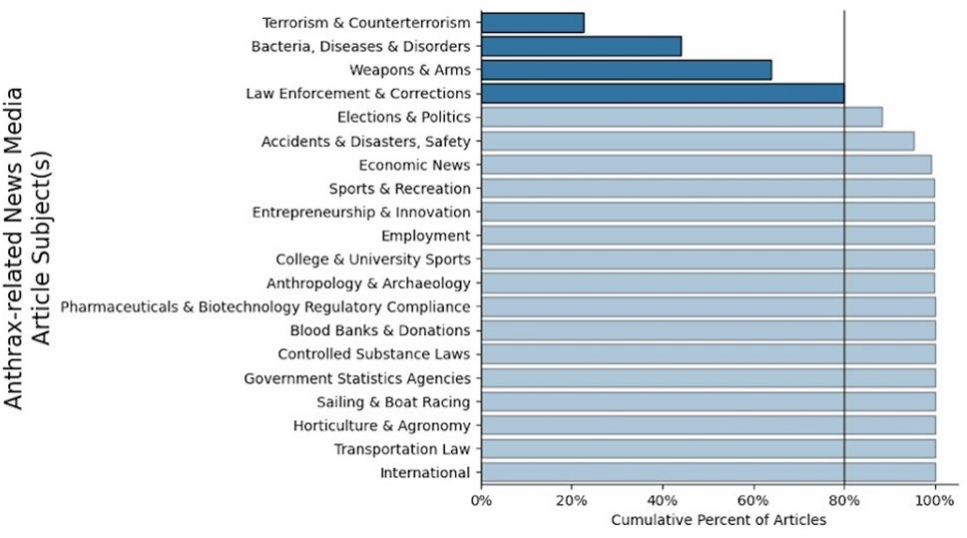
Cumulative percent of platform-identified anthrax-related news media articles associated with the twenty subject communities present in the sample of 20,054

We inspected the graphical representation of the subjects associated with each of the 20 identified communities and manually assigned a label for each community that we felt captured the major theme of the assigned subjects (Fig. 3). The four largest communities were associated with approximately 80% of the articles and focused on (A) terrorism & counterterrorism, (B) bacteria, diseases & disorders, (C) weapons & arms, and (D) law enforcement and corrections. The 80% significance threshold is standard in Pareto analyses (i.e., 80/20 rule). In our application, we identify the set of “vital” communities associated with the majority of discourse in the news media.

**Fig. 4:**
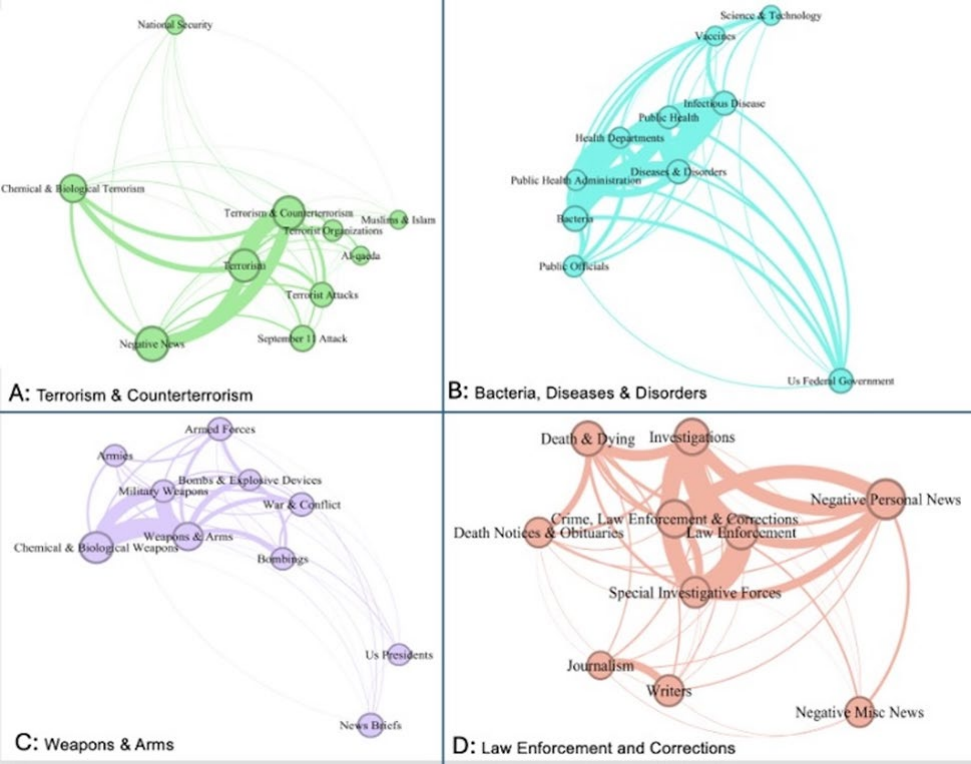
Graphical representation of the four key communities of subjects

The eigenvector centrality and connectivity of the nodes within the largest four subject communities provided a novel contextual background and meanings for stigmatization (Fig. 4). The “weapons & arms” community (Fig. 4, C) is situated with key nodes like “armed forces,” “bombs & explosive devices” and “chemical & biological weapons,” signaling a connection between bioterrorism and traditional warfare, integrating them within a militarized approach to terrorism. The “law enforcement and corrections” community (Fig. 4, D) summarized criminal investigations and personal impacts associated with anthrax, centering on nodes such as “investigations,” “negative personal news,” and “crime, law enforcement & corrections.” This community articulated law enforcement efforts with human-interest news media, including procedural work and personal tragedies. The “bacteria, diseases & disorders” community (Fig. 4, B) presented anthrax as an attack and public health crisis, linking infectious diseases with federal responses and emphasizing the importance of science and innovation. Finally, the “terrorism and counterterrorism” community (Fig. 4, A) tied anthrax to national security concerns in the post-9/11 context, framing it within bioterrorism and broader terrorism discourse.

### Variation in thematic content over time

**Fig. 5:**
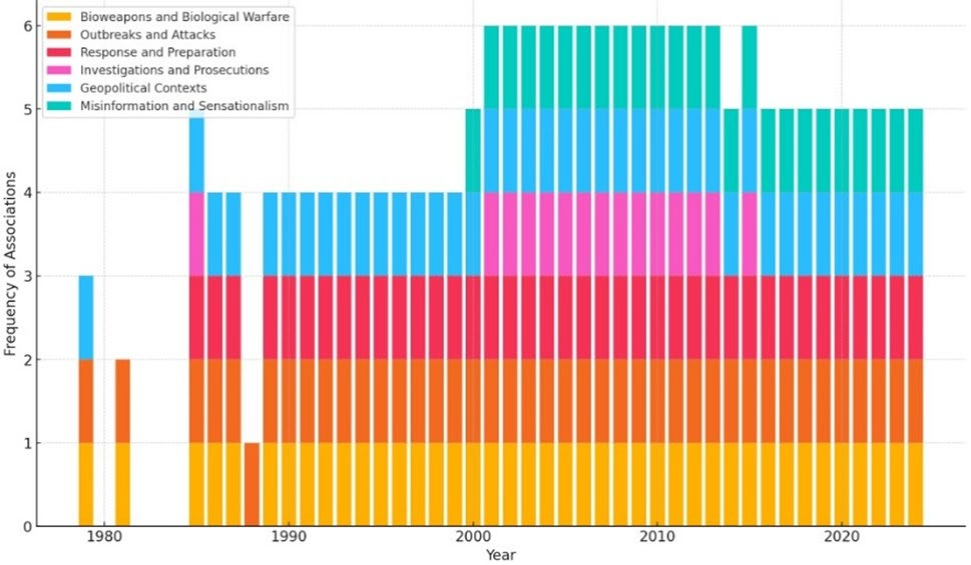
Temporal variation of thematic blocks. This chart illustrates the temporal variation in the frequency of associations with anthrax over the years, categorized by thematic blocks. The frequency of associations refers to the number of times specific terms related to each thematic block were mentioned in a given year. The Y-axis represents the number of associations identified per year, with a scale ranging from 0 to 6, indicating the maximum number of thematic blocks mentioned in a single year. This scale shows how many thematic categories were simultaneously present in the discourse or reviewed documentation. The 1980s are characterized by some data gaps, likely due to limited availability of information or lower prevalence of discussions regarding anthrax-related topics during that period. This is reflected in the absence or low frequency of associations in those specific years

NLP analysis identified six thematic blocks about anthrax-related news media coverage in the study sample, highlighting changes in stigmatization themes over 45 years. The six blocks were (in alphabetical order): (1) bioweapons and biological warfare, (2) geopolitical contexts, (3) investigations and prosecutions, (4) misinformation and sensationalism, (5) outbreaks and attacks, and (6) response and preparation. Each block was associated with terms that varied over time (Supplementary Table 2). This provided the basis for a more detailed temporal analysis of the thematic blocks related to anthrax-related news media coverage (Fig. 5).

Temporal analysis of thematic blocks identified several evolving patterns in the stigmatization of anthrax in news media articles, including the co-emergence of “response and preparation” and “investigations and prosecutions” in the late 1980s and “misinformation and sensitization” in the early aughts. Two thematic blocks, “bioweapons and biological warfare” and “outbreaks and attacks,” have remained consistent across the study period. Only one, “investigations and prosecutions,” has disappeared for an extended time before re-emerging.

## Discussion

This research advances insight into the stigmatization of public health issues in the news media, such as anthrax in the U.S., via a novel exploration of the subjects’ associations and the emergence and evolution of thematic blocks in news media articles over 45 years. The methodological approach of combining classical communication theories with advanced data science tools has demonstrated significant value to the analysis. It allowed for a more precise mapping of thematic evolution and media strategies over time, providing deeper insights into how media narratives were constructed and sustained. By bridging classical communication theories with modern data analysis, the study confirms theoretical arguments and expands our understanding of the intricate mechanisms contributing to stigmatizing health issues. Below, we discuss the most noteworthy findings from the research within the context of the extant literature, highlighting implications for public health practice.

### Militarization, sensationalism and misinformation of public health discourse

At least three related and noteworthy findings from this research are associated with the stigmatization of public health discourse related to militarization, sensationalism, and misinformation. In the case of anthrax, the media’s framing within the “weapons & arms” and “terrorism and counterterrorism” thematic communities (Fig. 4, C and A) illustrates the mechanisms by which anthrax has been embedded in a framework of bioterrorism. One key mechanism is the militarization of discourse, where anthrax was persistently associated with bioweapons, chemical warfare, and terrorism. This aligns with [27] argument that militarization transforms social relevant issues into matters of security, encapsulating them within a narrative of warfare. In this framing, anthrax was constructed as a threat to national security, imbuing it with the fear and danger typically associated with military conflict. This framing of anthrax as part of a more extensive arsenal of conflict solidified it as a symbol of bioterrorism. Like other military threats, it became shrouded in secrecy, fear, and avoidance in public discourse. Fear-driven language and its association with sensationalism and misinformation, particularly since the year 2001, have intensified public anxiety and hindered rational discourse, strengthening the perception of anthrax as a highly sensitive topic. According to Framing Theory [13], the media selects specific angles to present issues, often shaping how the public perceives these topics.

Social Amplification of Risk Framework further explains this by highlighting how the media can amplify certain risks, making anthrax appear more threatening than it might otherwise be, thus contributing to its stigmatization [24]. Another significant mechanism is the coupling of anthrax with themes of law enforcement and national security, as shown in Figs. 4, D and A. The media framed anthrax incidents as part of broader criminal investigations and counterterrorism efforts, embedding the topic within narratives of threat, justice, and defense. This framing often employs fear-evoking language—words like “weapons,” “attacks,” and “terrorism”—which constricts public engagement to being reactive and fear-based response [1, 16]. Instead of fostering open dialogue, this narrative confines anthrax to specialized, controlled discussions among law enforcement, military, and political leaders, further alienating it from everyday public discourse.

Furthermore, the symbolic association of anthrax with broader historical events, particularly post-9/11 terrorism and warfare, plays a central role in reinforcing its stigmatization. As both Goffman [17] and Furedi [16] suggest, linking an issue to traumatic national events magnifies societal fears. By repeatedly tying anthrax to national trauma and geopolitical conflict, the media constructed a narrative that made anthrax inseparable from larger societal anxieties. This symbolic linkage transformed anthrax from a biological zoonotic agent into an emblem of broader existential threats, branding it as a sensitive topic associated with vulnerability, insecurity, and fear.

### Diachronic analysis of stigmatization

Our diachronic analysis of stigmatization helps advance our understanding of factors that have influenced evolution. Results revealed clear consistency and continuity in the development of word blocks over the past 45 years. One of the primary mechanisms by which the news media constructs stigmatized subjects is the recurrence of specific themes over time, which helps naturalize particular narratives and embed them in public consciousness [9, 30]. For example, the theme block “bioweapons and biological warfare” maintained a consistent presence in news media articles, reinforcing anthrax as a recurrent global threat and bioterrorism risk. Consistent repetition is known to reinforce stigmatizations; in this case, the perception of anthrax as a danger, normalizing fear and creating an atmosphere where such threats are seen as inevitable [1]. The thematic block of “geopolitical contexts” was also persistent in demonstrating how public health concerns such as anthrax have been reinforced alongside changes in global power dynamics. Interestingly, however, stigmatization within this thematic block evolved to illustrate anthrax as an instrument of international conflict [4].

The sudden prominence of theme blocks during key moments in time is a strategy the news media can use to create or amplify the perception of urgency and crisis, a method well-documented in agenda-setting literature [6]. In our study, new theme blocks developed with very high visibility after the anthrax attacks of the early aughts (e.g., “investigations and prosecutions” and “response and preparation”). This development heightened coverage, framing anthrax not only as a biological problem but as a criminal and national security issue. Processes of thematic intensification can legitimize governmental actions and institutional responses, such as criminal investigations and security measures, contributing to narratives centered upon supporting state action and overt public safety activities [36]. At times, the development of stigma may result in dramatically elevated risk perceptions and be associated with alarmist language that generates an environment of fear and limits authentic debate [18, 29]. Sensational amplification can emerge as a key mechanism in constructing stigma, where the media magnifies the danger and creates barriers to open discussion and scientific analysis [23, 42]. The rise of the “misinformation and sensationalism” thematic block in the news media article dataset following 2001 helps underscore this potential for news media to help create moral panics from stigma.

This study highlights the potential beneficial opportunities of media communication in managing public health crises, particularly those involving societal fears such as bioterrorism. Such an approach would contribute to a more resilient public that is better equipped to navigate crises without the escalation of unnecessary panic or stigma. Ultimately, transparent communication could strengthen trust in public health systems and promote long-term social stability. By fostering accurate and balanced reporting, governments and health authorities could enhance public understanding, resulting in more informed and rational responses to health threats. Additionally, improving the management of information on neglected zoonotic diseases like anthrax would help allocate appropriate research and resources, recognizing its ongoing public health relevance beyond its association with bioweapons.

While the dataset used in this research captures a broad range of mainstream media coverage, incorporating additional perspectives from alternative sources could further enrich the analysis. Finally, we recognize opportunities for further exploration, such as incorporating social media sources, which have become a leading platform for news consumption. As public engagement shifts more completely from traditional to digital social media, this evolving landscape offers a chance to reassess and understand the current extent of media influence, which may differ from previous decades.

## Conclusions

This study highlights how media coverage has contributed to the stigmatization of anthrax by framing it predominantly as a bioterrorism threat rather than as a neglected zoonotic disease. By analyzing 20,054 news articles over 45 years, we identified key thematic shifts that reinforced fear-driven narratives and associated anthrax with national security concerns. These findings underscore the need for a more balanced media approach that avoids amplifying fear and instead fosters public understanding of anthrax as a broader public health issue. Addressing this stigma through accurate and contextualized reporting could improve risk communication strategies and public health interventions, ultimately reducing misinformation and promoting informed decision-making.

## Supplementary Information

Supplementary Material 1

Supplementary Material 2

## Data Availability

The raw media articles supporting the findings of this study are available in the University of Maryland, College Park repositories. The analysis data are stored in the University of Alabama repositories. These datasets include the unprocessed text files of the analyzed media articles downloaded from Nexis Uni™, the results of the subject communities analysis, and the frequency tables of the thematic blocks. Intermediate steps of the analyses are provided in the supplementary materials. Any additional data related to this article can be requested from the corresponding author.

## Abbreviations

NLP: Natural Language Processing
TF-IDF: Term Frequency-Inverse Document Frequency

## References

[1] Altheide DL. Terrorism and the Politics of Fear. Lanham: AltaMira Press; 2006.

[2] Andersson GZ, Reinius M, Eriksson LE, Svedhem V, Esfahani FM, Deuba K, Rao D, Lyatuu GW, Giovenco D, Ekström AM. Stigma reduction interventions in people living with HIV to improve health-related quality of life. Lancet HIV. 2020;7(2):e129–40. 10.1016/s2352-3018(19)30343-1. 31776098 10.1016/S2352-3018(19)30343-1 PMC7343253

[3] Barandongo ZR, Dolfi AC, Bruce SA, Rysava K, Huang YH, Joel H, Hassim A, Kamath PL, van Heerden H, Turner WC. The persistence of time: the lifespan of Bacillus anthracis spores in environmental reservoirs. Res Microbiol. 2023;174(6):104029. 10.1016/j.resmic.2023.104029. 36720294 10.1016/j.resmic.2023.104029

[4] Baumgartner FRJBD. Agendas and Instability in American Politics. Chicago: University of Chicago Press; 2009

[5] Bovet A, Makse HA. (2022). Centralities in Complex Networks. In B. Chakraborty, editor, Statistical and Nonlinear Physics (pp. 599–609). Springer US. 10.1007/978-1-0716-1454-9_765.

[6] Boydstun AE. Making the News: Politics, the Media, and Agenda Setting. Chicago: University of Chicago Press; 2013.

[7] Cairney P. The politics of evidence-based policy making. Palgrave Communications. 2016;2;16092.

[8] Carlson CJ, Kracalik IT, Ross N, Alexander KA, Hugh-Jones ME, Fegan M, Elkin BT, Epp T, Shury TK, Zhang W, Bagirova M, Getz WM, Blackburn JK. The global distribution of Bacillus anthracis and associated anthrax risk to humans, livestock and wildlife. Nat Microbiol. 2019;4(8):1337–43. 10.1038/s41564-019-0435-4. 31086311 10.1038/s41564-019-0435-4

[9] Carvalho A. Media(Ted) Discourse and Society: Rethinking the Framework of Critical Discourse Analysis. Journalism Stud. 2007;8(2):161–77.

[10] Day TG. The Autumn 2001 Anthrax Attack on the United States Postal Service: The Consequences and Response. J Contingencies Crisis Manage. 2003;11(3):110–7. 10.1111/1468-5973.1103004.

[11] Dimitrov R, Jelen A, L’Etang J. Taboos in health communication: Stigma, silence and voice. Public Relations Inq. 2022;11(1):3–35. 10.1177/2046147x211067002.

[12] Ellis R. Creating a secure network: the 2001 anthrax attacks and the transformation of postal security. Sociol Rev. 2014;62(1):161–82. 10.1111/1467-954X.12128.

[13] Entman RM. Framing: Toward clarification of a fractured paradigm. J Communication. 1993;43(4):51–8.

[14] Foucault M. The History of Sexuality. New York: Pantheon Books; 1978.

[15] Freimuth V. Epilogue to the Special Issue on Anthrax. J Health Communication. 2003;8(sup1):148–51. 10.1080/713851979.

[16] Furedi F. Invitation to Terror: The Expanding Empire of the Unknown. New York: Continuum; 2007.

[17] Goffman E. Stigma: Notes on the Management of Spoiled Identity. Englewood Cliffs: Prentice-Hall; 1963.

[18] Goode EB-YN. Moral Panics: The Social Construction of Deviance. Wiley-Blackwell; 2009.

[19] Halldorsson H. The overwhelming case for ending stigma and discrimination in mental health. 2024 https://www.who.int/europe/news/item/26-06-2024-the-overwhelming-case-for-ending-stigma-and-discrimination-in-mental-health.

[20] Handysides S. The History of Bioterrorism: Old Idea, New Word, Continuing Taboo. In: Lutwick L, Lutwick S, editors. Beyond Anthrax. The Weaponization of Infectious Diseases. New York: Springer. 2009;1–16.

[21] Hswen Y, Xu X, Hing A, Hawkins JB, Brownstein JS, Gee GC. Association of #covid19 Versus #chinesevirus With Anti-Asian Sentiments on Twitter: March 9–23, 2020. Am J Public Health. 2021;111(5):956–64. 10.2105/ajph.2021.306154. 33734838 10.2105/AJPH.2021.306154 PMC8034032

[22] Joffe H, Haarhoff G. Representations of far-flung illnesses: the case of Ebola in Britain. Soc Sci Med. 2002;54(6):955–69. 10.1016/s0277-9536(01)00068-5. 11996028 10.1016/s0277-9536(01)00068-5

[23] Kasperson RE, Jhaveri N, Kasperson JX. Stigma and the Social Amplification of Risk: Toward a Framework of Analysis. In: Flynn J, Slovic P, Kunreuther H, editors. Risk, media, and stigma: Understanding public challenges to modern science and technology. New York: Earthscan. 2001;9–30.

[24] Kasperson RE, Renn O, Slovic P, Brown HS, Emel J, Goble R, Kasperson JX, Ratick S. The Social Amplification of Risk: A Conceptual Framework. Risk Anal. 1988;8(2):177–87. 10.1111/j.1539-6924.1988.tb01168.x.

[25] Lu Y, Kaushal N, Huang X, Gaddis SM. Priming COVID-19 salience increases prejudice and discriminatory intent against Asians and Hispanics. Proceedings of the National Academy of Sciences. 2021;118(36):e2105125118. 10.1073/pnas.2105125118. 10.1073/pnas.2105125118 PMC8433560 34462353

[26] Lutwick L, Lutwick S, editors. Beyond Anthrax. The Weaponization of Infectious Diseases. New York: Springer; 2009.

[27] Lutz C. Making War at Home in the United States: Militarization and the Current Crisis. Am Anthropol. 2002;104(3):723–35. 10.1525/aa.2002.104.3.723.

[28] Mableson HE, Okello A, Picozzi K, Welburn SC. Neglected zoonotic diseases-the long and winding road to advocacy. PLoS Negl Trop Dis. 2014;8(6):e2800. 10.1371/journal.pntd.0002800. 24901769 10.1371/journal.pntd.0002800 PMC4046968

[29] Morin CFDJ. Misinformation and the justification of social and policy responses to COVID-19. J Health Communication. 2022;27(5):376–86.

[30] O’Neill KM, Calia JM, Chess C, Clarke L. Miscommunication during the Anthrax Attacks: How Events Reveal Organizational Failures. Hum Ecol Rev. 2007;14(2):119–29. http://www.jstor.org/stable/24707698.

[31] Oliver K, Innvar S, Lorenc T, Woodman J, Thomas J. A systematic review of barriers to and facilitators of the use of evidence by policymakers. BMC Health Serv Res. 2014;14:2. 10.1186/1472-6963-14-2. 24383766 10.1186/1472-6963-14-2 PMC3909454

[c32] Sellnow TL, Ulmer RR, Seeger MW, Littlefield RS. Effective Risk Communication: A Message-Centered Approach. New York: Springer; 2009.

[33] Shannon G, Jansen M, Williams K, Cáceres C, Motta A, Odhiambo A, Eleveld A, Mannell J. Gender equality in science, medicine, and global health: where are we at and why does it matter? Lancet. 2019;393(10171):560–9. 10.1016/s0140-6736(18)33135-0. 30739691 10.1016/S0140-6736(18)33135-0

[34] Sparck Jones K. A Statistical Interpretation of Term Specificity and its Application in Retrieval. J Doc. 1972;28(1):11–21. 10.1108/eb026526.

[35] Steffan JJ, Derby JA, Brevik EC. Soil pathogens that may potentially cause pandemics, including severe acute respiratory syndrome (SARS) coronaviruses. Curr Opin Environ Sci Health. 2020;17:35–40. 10.1016/j.coesh.2020.08.005. 33521411 10.1016/j.coesh.2020.08.005 PMC7836926

[36] Stritzel H. Securitization, power, intertextuality: Discourse theory and the translations of organized crime. Secur Dialogue. 2012;43(6):549–67. 10.1177/0967010612463953.

[37] Suhay E. The politics of science: Political values and the production, communication, and reception of scientific knowledge. J Health Communication. 2013;18(12):1422–37.24083417

[38] Swain KA. Outrage Factors and Explanations in News Coverage of the Anthrax Attacks. Journalism Mass Communication Q. 2007a;84(2):335–52. 10.1177/107769900708400209.

[39] Swain KA. Sourcing Patterns in News Coverage of the Anthrax Attacks. Int J Mass Emergencies Disasters. 2007b;25(1):57–96. 10.1177/028072700702500103.

[40] Traag VA, Waltman L, van Eck NJ. From Louvain to Leiden: guaranteeing well-connected communities. Sci Rep. 2019;9(1):5233. 10.1038/s41598-019-41695-z. 30914743 10.1038/s41598-019-41695-z PMC6435756

[41] Villa S, Jaramillo E, Mangioni D, Bandera A, Gori A, Raviglione MC. Stigma at the time of the COVID-19 pandemic. Clin Microbiol Infect. 2020;26(11):1450–2. 10.1016/j.cmi.2020.08.001. 32777361 10.1016/j.cmi.2020.08.001 PMC7411378

[42] Vosoughi SRDAS. The spread of true and false news online. Science. 2018;359(6380):1146–51. 29590045 10.1126/science.aap9559

