# Supplementary material for: Stigmatizing bioterrorism as a public health concern: The case of anthrax in US media
Source: BMC Public Health. 2026 Mar 6;26:1202. doi: 10.1186/s12889-026-26851-1 (PMC13077897; doi:10.1186/s12889-026-26851-1)
Supplement: Supplementary file 1 — Supplementary Material 1 [file 12889_2026_26851_MOESM1_ESM.docx]

**Supplementary Table 1.** Distinguishing keywords or phrases by year based on the technique Term Frequency-Inverse Document Frequency (TF-IFD).

| **Years** | **Significant keywords or phrases** |
| --- | --- |
| 1979 | disease death humans animals, 30 plus, 1979 plume, facility soviet city sverdlovsk, secret bioweapons facility, called biological, outbreak inhalation anthrax, anthrax spores accidentally released secret bioweapons, people lost lives, plume anthrax spores, biological chernobyl, south east, 66 people, april 1979, education secretary, education project said, edward montooth, education research, edward ivins |
| 1981 | including number, chinese russian, infection developed, prisoners died, botulism brucellosis, symptoms incubation period, human casualties, number people infected, incubation period seven days, autopsies performed, scientific information, result experiments, infected number, lethal dosages, russian american, glanders smallpox, assembling biological, used human guinea pigs, massive doses, number human, disease aerosol, day report, determine lethal, tests including, described anthrax, plague typhus, human animal plant, number died, result massive, anthrax experiments, spreading disease, gas gangrene, died result, anthrax tularemia, cholera anthrax |
| 1985 | arms control, causing epidemic, peace movement, united states created, long standing biological, weapons acquired, anthrax inadvertently, biological weapons stocks, said hands, released 1979, according pentagon, anthrax difficult control, deadly soviet, reported soviet, washington centre, department destroyed, politically militarily, scary according, loophole treaty, research genetic engineering, repeated accusations, highly pathogenic organisms, say reported, agents anthrax difficult, government report said, according robert, mikhail gorbachev, 1979 soviet biological, report said anthrax, centre strategic international studies, forces used, destroyed biological weapons, funding research, troops according, hands government, scientists questioned, book called, western scientists, says outbreak, says work |
| 1986 | yellow fever anthrax plague, germs including, highly dangerous, far greater, anthrax plague botulism, dangerous germs, said army, edward eitzen, education secretary, education research, education project said, effect anthrax attacks, edward ivins, education military, education initiatives, education department, educating doctors, edward george, edward kennedy mass, educate people, edward lake retired computer, edward montooth, edwards ivins, edwards said, eerily reminiscent, eerily similar, effect american, educate public, editorial statement lack training force received, editor sun supermarket tabloid boca raton, editor sun supermarket tabloid published american, editor sun tabloid, editor supermarket tabloid sun died |
| 1987 | anthrax 1979, airborne anthrax, sverdlovsk city, scientists experimented anthrax, immediate future, bacteria carried, hundreds civilians, disease better, pushed wings, like death, anthrax readers, drop bombs, spy satellites, according defence department, public record office kew, insisted outbreak, success comes, titles like, bacteriological war, anthrax smells, soviets insisted, october 19, weapons ban, better suited, premier mike, war did, word association, conduct war, anthrax best seller, anthrax bombing, known british, clean efforts, said soviets, island gruinard north west coast scotland, poll tax, officials said airborne, escape anthrax, civilians infected, island decontaminated, yesterday stories, production delays |
| 1988 | cause anthrax infection skin lungs, skin lungs digestive tract, bad weather, days released, bacilli cause, turn spores, dormant decades, island just, kill man, gruinard island, biological warfare agent, anthrax bacillus, sheep cattle, anthrax control, fever makes, affects human beings, bacterium known bacillus anthracis, anthrax spores took notes, biological defense research, said camp, known wool sorter disease, pushed wings, october 1987, spreads cause, skin bacterium, acknowledged people, researchers exploded canisters containing billions anthrax, claimed incident, ago test, refer anthrax, affect tens thousands |
| 1989 | cholera typhoid anthrax, black death, containing anthrax bacteria cause, israeli foreign, yesterday threat, allowed happen, disease involved, people experience, arrived britain, guarantee security, produced bacterial, diseases cholera anthrax, pure anthrax, bombs containing anthrax bacteria, symptoms black, general shiro ishii unit, abc said, unit helped, denied yesterday, security israeli, people watched, include biological, plague anthrax diseases, spread lethal diseases, developing capacity, research weapons, infecting large, wells rivers, anthrax paratyphoid, ishii men, west country, bacterial cultures, developing deadly, diseases like cholera, spreading plague anthrax, assembling biological, badly hit, country probably, plague virus, man charge |
| 1990 | multinational force, saudi arabia, years iraq, iraq developed, biological agents including botulism anthrax, attack aircraft, ground troops, intelligence agencies evidence, evidence years, iraq conducting, iraqi invasion, developed powerful, botulism anthrax typhoid, force likely, war attrition, rotten meat, cholera tularemia, usually fatal, protection biological weapons, killing fields, electronic warfare, ground forces, american army, powerful strain, anthrax typhoid cholera, young daughters, yellow rain, united states, anthrax eating, forces gulf, western intelligence agencies, counter attack, biological arsenal, anthrax airborne, iraq developing, oil fields, troops inoculated, 10 years ago, public opinion, equine encephalitis, conducting research, said iraq |
| 1991 | allied troops, end result, saddam hussein, 52 bombers, agents says, iraqi arsenal, quoted egyptian, iraqis use, soldiers died, chemical suits, biological agents anthrax botulinum toxin, germ warfare, gas biological, iraqi soldiers, diseases anthrax botulism, reports iraq, west nile fever, saudi arabia, troops inoculated, western intelligence, arab world, american troops, nerve gases, use anthrax, chemical biological agents, anthrax vaccine, gas masks, use biological weapons, warfare agents, iraq conducting, contaminated animal hides, nervous biological agents, mustard gas tons, days vaccine, raf officer, american association advancement science |
| 1992 | mysterious outbreak anthrax, know refuse, sharp eyed, developing strains, iraq biological weapons arsenal, anthrax poisoning area, doctor probably, black spot, security situation, told interviewer, special health, kind britain, believed case, biological weapons general, hepatitis hepatitis, experience guerrilla, insurance payment, interview anthrax, anthrax farmer, lot work just, week interview, compensate victims, anthrax friends, won know, probably won, plague rabies anthrax, guard said, london yesterday, causing delays, case kind, admit ignorance, worst moment, friends told, 60 people died, vaccines exist, minister defence, military activity, week told, use war, potentially deadly disease anthrax, biological capability, similar reports, life insurance, fever bubonic plague, electronic mail, anthrax near |
| 1993 | vaccinations anthrax, certain conditions, national leaders, died anthrax look, week monday, group public citizen, world order, words panic, easy treat, used soldiers, dismissed british, half expecting, anti nerve gas pills, left man, develop infected, gen ronald blanck, employ biological, said anthrax spread, immediately war, recover anthrax, short breath, possibility iraqi, walter reed army medical, come left, public group, response threat anthrax, personally killed, threat anthrax vaccine, forces employ, anthrax tetanus spores, typhoid bubonic plague, diphtheria cholera, intelligence reports indicated, animal died anthrax, humans catch, routine vaccinations, anthrax outbreaks anthrax, anthrax 24 hours, animal dies anthrax, infected humans, cow dead |
| 1994 | military compound, immunizations anthrax, uw madison, chocolates laced, carried wind, biological weapons, tainted meat, biological warfare, anthrax deaths, bubonic plague plague, april 1992, anthrax poisoning killed, deliberate contamination, smoke burning, right expect, died given, shadow cabinet, gorbachev glasnost, meselson team, anthrax blair, treaty biological, gave children, bubonic plague anthrax whooping cough, month 500, miles east moscow, accident occurred june, war possible biological, mikhail gorbachev, way wind blowing, communist party chief, suffering dying, photographs showed, killed 66 people russian, antidote biological, anthrax investigated, releasing anthrax spores, said aggressively, coast island gruinard, anthrax spores released air, willing accept, eradicated united states |
| 1995 | anthrax whooping cough, gulf war, dr murphy, carriers anthrax, determine nation, suitable production, germs grown, antibiotics ve, plague organisms, grow germs, production anthrax botulinum, anthrax botulinum plague, antibiotics developed, nation preparing, dr germs, medical profession, virulent anthrax strains, trying buy, anthrax bubonic plague, says report, anthrax botulism, spore form, yellow fever, weapons inspectors, missile warheads, spreading anthrax, staphylococcus aureus, australian medical association, subway systems, species barrier, botulism gas gangrene, germ warfare weapon, deadly perfume, military office, london paris, malcolm rifkind, doctors finding, weapons biological warfare |
| 1996 | biological weapons, israeli sources, scientists according, gulf war, anthrax botulin, newsday reported, rockville md company, according congressional records, fever biological, pentagon study, sheep goat, toxin fever, produce stocks, stocks quickly, director company exported, stocks anthrax botulism, company 70 government approved shipments anthrax, aum supreme truth, iraq 1991 war, anthrax bacilli, pathogens iraqi scientists, religious sect, arsenal included, bubonic plague, nuclear warhead, 1985 1989 |
| 1997 | anthrax spores, anthrax bacteria kill, biological weapons, gulf war, anthrax botulism, weapons inspectors, days 100, deadly biological, kill tens thousands people, anthrax deadly, germ warfare, millionth gram anthrax, tiny particles, saddam hussein, biological warfare, scud missiles, persian gulf, chemical biological, 10 countries, weapon anthrax, anthrax iraq, bag sugar, secretary william cohen, anthrax botulinum toxin, nerve gas, biological agents, warfare iraq, afflicts cattle, chlostridium perfringens, deadly anthrax, warfare agent, spores anthrax, anthrax outbreak, gallons anthrax, 150 000, 100 000 people |
| 1998 | duty free, las vegas, biological weapons, saddam hussein, deadly anthrax, anthrax spores, nerve gas, smuggle anthrax, saddam anthrax, 000 gallons, chemical biological, chemical weapons, million people, gallons anthrax, united states, wipe city, anthrax vaccine, anthrax bacteria, tel aviv, usually afflicts, botulinum toxin, disease usually, larry wayne harris, george robertson, germ warfare, mass destruction, home office, deliver anthrax, military grade anthrax, defence secretary, persian gulf, 000 chemical, terry taylor, afflicts animals especially cattle sheep anthrax, air sea, weapons mass |
| 1999 | anthrax vaccine, anthrax spores, biological weapons, smallpox anthrax, gulf war, anthrax smallpox, said col, anthrax shots, nerve gas attack, gerald parker, marine corps, anthrax vaccinations, days later, pfc james neil, dead local, service members, rush hour, smallpox highly, deadly anthrax, parker army, just exposed anthrax, doctors believe, soon exposure, anthrax scenario, flu days, anthrax plague, anthrax program, abortion clinics, anthrax threats, antibiotics prevent anthrax, 300 people, unusual symptoms, united states, million people, anthrax attack, said pointing, far affected, roswell said, cause terror, number anthrax hoaxes, botulism toxin anthrax, carr said, biological warfare |
| 2000 | anthrax vaccine, biological weapons, infection initially, united states, initially led, inhaled anthrax, nerve gas, plague nerve, nerve agent sarin gas, believe illness, bacterial agents anthrax plague, deadly bacterial agents, people exposed, caused anthrax, biological agents, formally approved, treat deadly, bayer corp, health officials, illness caused, advisory committee, smallpox anthrax, anthrax spores, gulf war, air force, anthrax tests confirmed, botulism toxin anthrax, said cipro, highly secret, deal government, anthrax brucella, biological warfare, soviet union, variety infections, cattle horses, anthrax botulism, planned parenthood, terrorists using, release anthrax, outbreaks disease |
| 2001 | new york, anthrax attacks, anthrax spores, sept 11, inhalation anthrax, united states, new jersey, anthrax cases, american media, postal workers, anthrax scares, health officials, terrorist attacks, officials said, letter sent, skin anthrax, traces anthrax, white house, osama bin laden, senate majority leader tom daschle, tested positive anthrax, september 11, form anthrax, form disease, boca raton, postal service, year old, exposed anthrax, public health, cases anthrax, tom brokaw, daschle office, capitol hill, disease control, post office |
| 2002 | sept 11, anthrax spores, september 11, new york, anthrax letters, united states, biological weapons, fall anthrax attacks, dr hatfill, anthrax scares, fort detrick, anthrax smallpox, 11 attacks, killed people, year anthrax attacks, postal service, officials said, public health, attacks anthrax, anthrax laced, 11 terrorist attacks, steven hatfill, attacks fall, tom daschle, anthrax investigation, new jersey, deadly anthrax, anthrax mailings, anthrax vaccine, postal workers, law enforcement, chemical biological, centers disease control, biological agents, laced letters, 11 anthrax, ames strain anthrax, botulinum toxin |
| 2003 | biological weapons, sept 11, weapons inspectors, anthrax vx nerve, anthrax smallpox, vx nerve agent, october 2001, anthrax spores, united states, anthrax scare, chemical biological, 000 litres anthrax, postal service, botulinum toxin, liters anthrax, terrorist attacks, saddam hussein, anthrax vaccine, said iraq, september 11, weapons mass destruction, mustard gas, smallpox anthrax, new york, biological agents, hans blix, chemical weapons, vx nerve gas anthrax, dr blix, united nations, 2001 anthrax attacks, 25 000 liters, 10 000 litres, 11 2001, blix said |
| 2004 | 2001 anthrax attacks, anthrax vaccine, anthrax ricin, new york, anthrax spores, postal service, mrs anthrax, october 2001, terrorist attacks, united states, anthrax attacks 2001, known dr germ, capitol hill, sept 11 2001, huda salih mahdi ammash, public gallery, ricin anthrax, biological weapons, dr kenneth berry, anthrax mailings, anthrax smallpox, chemical biological, phial anthrax, al qaeda, smallpox anthrax, killed people, postal workers, agents investigating, majority leader, capitol police, rihab rashid taha, senate office, anthrax laced |
| 2005 | known dr germ, anthrax attacks, biotech researcher, baath party, huda salih mahdi ammash known mrs, salih mahdi ammash known mrs anthrax, expert known, rihab taha british, making bio, weapons 1980s, sept 11, public health, biological weapons expert, anthrax laced letters, 2001 anthrax, september 11, biodefense laboratory, mail facilities, bio weapons, 11 attacks, new york, anthrax letters, protection nerve gas, 11 2001 |
| 2006 | anthrax spores, mr hatfill, kristof columns, anthrax scare, killed people, sept 11, 2001 anthrax attacks, confidential sources, american media, new york times, united states, attacks anthrax, october 2001, al qaeda, nicholas kristof, september 11, postal service, capitol hill, terrorist attacks, ordered new, 11 attacks, 11 anthrax, federal judge, sources used, 11 2001, homeland security, steven hatfill, deadly anthrax mailings 2001, new anthrax vaccine, biological weapons, pascal norris, possible health, animal hides, mr kristof, public health, samples taken, series columns, unsolved anthrax, million americans |
| 2007 | - united states, new york, village hall, anthrax vaccine, anthrax scare, anthrax mailings, anthrax spores, mr norris, production biological weapons, anthrax attacks 2001, anthrax smallpox, dirty bomb, sept 11 2001, 11 attacks, american soil, anthrax attacks capitol hill, anthrax following, attacks anthrax, dr hatfill, 11 2001 terrorist attacks, 11 terrorist attacks, immunized anthrax, wounded soldier, 2001 anthrax attacks capitol, doesn anthrax, postal service refused, pretty easy, refused deliver mail, bird flu, october 2001 |
| 2008 | dr ivins, 2001 anthrax attacks, justice department, killed people, committed suicide, anthrax letters, anthrax mailings, ivins worked, anthrax laced letters, anthrax investigation, usa today reporter, scientist bruce ivins, anthrax vaccine, ivins 62, dr bruce ivins, new york, genetic mutations, fort detrick md, toni locy, dr hatfill, court documents, sept 11 2001, sources stories, said ivins, highly purified anthrax spores, anthrax used attacks, scientist steven hatfill, attacks killed, army scientist bruce |
| 2009 | dr ivins, terrorist attacks, bruce ivins, anthrax scare, isolated incident, bush administration, fort detrick, anthrax vaccine, united states, new york, experimental drug, anthrax spores, anthrax attacks 2001, 2001 anthrax attacks, anthrax letters, al qaeda, dr hatfill, sept 11 2001, deadly anthrax, 11 attacks, teenage accomplice 2002 sniper shootings killed, accomplice 2002 sniper shootings killed 10, 2002 sniper shootings killed 10 people, sniper shootings killed 10 people terrified, killed 10 people terrified washington, carrying teenage accomplice 2002 sniper shootings, masterminding carrying teenage accomplice 2002 sniper |
| 2010 | dr ivins, anthrax attacks, bruce ivins, drum circle, anthrax spores, anthrax letters, milk supply, mr ivins, capitol police, details case, mailed anthrax, sept 11, importance government, new documents, killed people, al qaeda, new importance, released fbi, mailings work, ivins troubled, fbi documents, newly released, time letters, efforts anthrax, work given, life work, prime minister, letters sent, ivins lab, government researcher, 11 attacks, united states, given new, anthrax scare, white house, case friday, dr heine, deadly 2001 anthrax mailings fbi, probing deadly, expert killed 2008, block writing, notes mailed, new york, justice department, closed case |
| 2011 | dr ivins, fort detrick, sept 11, anthrax spores, anthrax letters, new york, justice department, anthrax vaccine, al qaeda, 11 attacks, killed people sickened 17, 2001 anthrax attacks, anthrax mailings, anthrax powder, committed suicide, united states, medical research, robert stevens, mailed anthrax, briscoe said, letters containing, postal workers, sent mail, deadly anthrax, news organizations, report suspicious, federal government, anthrax scare, tabloid photo editor, dr bruce ivins, 11 2001, maureen stevens, homeland security, biological agents, biological weapons, public health, american media publisher national, government agreed pay |
| 2012 | anthrax spores, elephants died, stem cells, sept 11, 2001 anthrax attacks, al qaeda, coli bacteria, patrick said, inhibit anthrax, professor les baillie, tea ability, postal service, public health, containing anthrax, white powder, like anthrax, 11 2001, bacillus anthracis bacterium causes anthrax, spokesman said, terrorist attacks, agents sarin anthrax, middle east including, school pharmacy pharmaceutical sciences, government secret military research, anthrax long black, ability inhibit, tough coating, meat sick, heat radiation, largest stockpiles, industrial research, pakistan prime minister |
| 2013 | terrorist attacks, sept 11 2001, letters laced, parallels anthrax, new york, killed people, 2001 anthrax attacks, reminded anthrax attacks, tracking program created, anthrax spores, anthrax scare, letters sent, anthrax attacks wake september 11, shorehead shutdown, told reporters briefing, senators told, anthrax letters, wake september 11 2001 attacks, mail isolation, ricin laced, anthrax laced letters, laced anthrax, attacks sept 11, white house, attacks pentagon world trade |
| 2014 | anthrax attacks, centers disease control prevention, anthrax spores, human pathogens, mail fbi, 2001 anthrax, individuals access, bird flu, bruce ivins, sept 11, anthrax hold, died 42, dangerous anthrax bacteria, bid prevent, government biodefence, biosecurity regulations, 500 laboratories, rules require, like anthrax sars, scientist worked government, workers labs, united states letters laced anthrax, 11 2001, pathogens toxins, flu virus, security sensitive, regulations govern, letters laced anthrax began appearing mail, exposed died, terrorist attacks united states letters, civil surgeon, live anthrax |
| 2015 | base south korea, osan air base, live anthrax spores, united states, live samples, shipped live anthrax, white powder, anthrax attacks, live anthrax samples, mistakenly sent, dugway proving ground utah, sent live anthrax, department defense, potentially exposed, shipments live anthrax, commercial labs, labs usa, risk general public, 22 people, public health, army dugway proving ground, anthrax specimens, accidentally sent, live spores, envelopes containing, 2001 anthrax, foreign countries, labs working, officials said, lab workers, anthrax sent, army lab |
| 2016 | anthrax attacks, attack kenya, dangerous lethal, network included medical experts, planning large scale, public health agency canada, anthrax bacteria, michael mosley, anthrax spores, westgate mall, medical intern, kill anthrax specimens, exposed anthrax, 2001 anthrax, largely forgotten, unleash biological, expert bioterrorism, police chief, delivered various, large scale attacks, biological attack using anthrax, biological weapons, shipping live anthrax, 70 years ago, sept 11, wide range, research facility, abortion services |
| 2017 | - north korean, cheong wa dae, biological weapons, http www, kim jong, anthrax attacks, anthrax vaccines, presidential office, anthrax smallpox, lethal version, united states, https www, sept 11, korea centers disease control prevention, islamic state, white powder, doses anthrax, load anthrax, chemical weapons including anthrax, biological agents, antimicrobial peptides, tonnes chemical weapons including, 000 tonnes chemical weapons, white paper, anthrax intercontinental ballistic missiles, anthrax clostridium botulinum, south korean military, korea institute |
| 2018 | british troops vaccinated anthrax, prince harry, defence secretary, thousands british troops, gavin williamson, anthrax scare, kensington palace, high readiness, kim jong, anthrax spores, st james, 2001 anthrax attacks, white powder purporting anthrax, letter containing white powder, anthrax vaccine, defence centre, nerve agent, thousands troops, new chemical, war north, chemical weapons defence, ricin anthrax, north korean leader, royal family, killed people, 2001 letters containing |
| 2019 | - white powder, marked anthrax, hoax white, packages marked, powder cases, anthrax female, welsh minister rhodri morgan, envelopes marked, wales 2002, kill person earth, anthrax attacks, north korean, sent hoax anthrax, hoax anthrax mail, mail court, biological weapons, botulism deadly, way kill, united states, court heard, jailed years, acquire anthrax, flu botulism, able acquire, jeanne guillemin, spanish flu, ebola smallpox anthrax, court papers, donald trump, coast guard, 500 years, american anthrax fear crime investigation nation, deadliest bioterror, anthrax fear crime investigation nation deadliest, anthrax spores stay |
| 2020 | covid 19, chinese anthrax, coronavirus outbreak, wuhan 400, test kits, coronavirus cases, united states, social distancing, coronavirus pandemic, confirmed cases, new coronavirus, spread coronavirus, anthrax chinese, empress anthrax, prime minister, death toll, spread virus, cases coronavirus, kadlec said, new cases, world war, new york, biological weapons, sinaloa cartel, march 17, total number, johns hopkins university, human body, state emergency, hong kong, stay home, 000 people, kadlec office, novel coronavirus, permanently contaminate objects, survive outside, public health labs, donald trump, south korea, sept 11, 2001 anthrax attacks |
| 2021 | zone anthrax, hot zone, reuters reports, national geographic, united states, anthrax vaccines, emergent anthrax, new york, pfizer biontech, european union, coronavirus pandemic, covid 19 vaccines, coronavirus vaccines, public health, biological weapons, sars cov, 11 attacks, money buy, strategic national stockpile, state michigan, paid emergent, half billion, health ministry, billion dollar, health minister, covid 19 pandemic, nearly half, september 11, kadlec said, ½ï ½s, 2001 anthrax attacks, letters containing, al qaeda, mr el hibri, government money, government spent, health officials |
| 2022 | anthrax attacks, pathogens plague anthrax, covid 19, biological laboratories, plague anthrax tularemia, plague anthrax cholera, biological weapons, united states, deadly diseases, african swine fever, dangerous pathogens, lethal diseases, seven presidents, deadly pathogens, particularly dangerous, fever cholera, anthrax island, rabbit fever, cures antidotes killers bubonic plague anthrax, devastating diseases, vector involved efforts cures antidotes killers, hiv cancer, years vector involved efforts cures, involved efforts cures antidotes killers bubonic |
| 2023 | changed way americans, covid 19, mailed packages, anthrax bomb dropped, united states, west coast, al shabaab, signs symptoms, 2001 anthrax attacks, bacillus anthracis, anthrax mailing, july 1995, burry inlet, funded research, ministry agriculture, watch tv, air travel, 11 attacks anthrax, contaminated animal products, risk exposure, contracting anthrax, health scares, years sept 11, anthrax spores deliberately, people sick anthrax come contact infected, lab leak, hiv sars mers ebola, come contact infected animals contaminated animal |
| 2024 | st petersburg, anthrax scare, skin anthrax, tests positive, iraq alleged weapons, daschle office, inhalation anthrax, howard troxler, sept 11, new jersey, anthrax officials, new york mayor rudolph, postmarked trenton sent, test positive anthrax, friday night, positive exposure, mobile biological weapons, senate majority leader tom daschle, house office building, 000 liters, big picture, hastert said, washington area postal workers, gov george pataki, 22 washington, testing thousands postal |
