# Supplementary material for: Stigmatizing bioterrorism as a public health concern: The case of anthrax in US media
Source: BMC Public Health. 2026 Mar 6;26:1202. doi: 10.1186/s12889-026-26851-1 (PMC13077897; doi:10.1186/s12889-026-26851-1)
Supplement: Supplementary file 2 — Supplementary Material 2 [file 12889_2026_26851_MOESM2_ESM.docx]

**Supplementary Table 2.** Nature Language Processing analysis identified six thematic blocks across 20,054 anthrax-related news media articles published over the past 45 years. Multiple word blocks and phrases (in alphabetical order) were associated with each thematic block and varied across years.

| **Thematic Block** | **Word Blocks or Phrases** | **Associated Years** |
| --- | --- | --- |
| Bioweapons and Biological Warfare | (n = 11) "biological agents," "biological arsenal," "biological capability," "biological warfare," "bioterrorism," "bioweapons," "chemical biological weapons," "chemical weapons," "developing strains," "germ warfare," "weapons inspectors." | 1979, 1981, 1985, 1986, 1987, 1990, 1991, 1992, 1993, 1994, 1995, 1996, 1997, 1998, 1999, 2000, 2001, 2002, 2003, 2004, 2005, 2006, 2007, 2008, 2009, 2010, 2011, 2012, 2013, 2014, 2015, 2016, 2017, 2018, 2019, 2020, 2021, 2022, 2023, 2024 |
| Geopolitical Contexts | (n = 12) "biological threat," "biological weapons inspectors," "geopolitical," "gulf war," "Iraq," "Iraqi arsenal," "military activity," "Russia," "Saddam Hussein," "Soviet city Sverdlovsk," "Soviet Union," "United States." | 1979, 1985, 1986, 1987, 1990, 1991, 1992, 1993, 1994, 1995, 1996, 1997, 1998, 1999, 2000, 2001, 2002, 2003, 2004, 2005, 2006, 2007, 2008, 2009, 2010, 2011, 2014, 2015, 2016, 2017, 2018, 2020, 2021, 2022, 2023, 2024 |
| Investigations and Prosecutions | (n = 12) "bioterrorism investigation," "Bruce Ivins," "court documents," "Dr. Hatfill," "Dr. Ivins," "FBI," "investigation," "investigations," "justice department," "prosecutions," "Steven Hatfill," "suspect." | 1985, 1990, 1991, 1992, 1993, 1995, 1996, 1997, 1998, 1999, 2000, 2001, 2002, 2003, 2004, 2005, 2006, 2007, 2008, 2009, 2010, 2011, 2014, 2016, 2020, 2021, 2022, 2023, 2024 |
| Misinformation and Sensationalism | (n = 13) "anthrax hoaxes," "hoax anthrax," "media," "media frenzy," "media reports," "news coverage," "newspaper," "public fear," "public panic," "public reaction," "scare," "sensationalism," "terrorist scare." | 2001, 2002, 2003, 2004, 2006, 2007, 2008, 2009, 2010, 2011, 2012, 2013, 2014, 2015, 2016, 2017, 2018, 2019, 2020, 2021, 2022, 2023, 2024 |
| Outbreaks and Attacks | (n = 14) "2001 anthrax attacks," "anthrax cases," "anthrax laced letters," "anthrax mailings," "anthrax scare," "anthrax spores," "attacks," "attacks anthrax," "biological attack," "letters laced," "outbreak," "public health," "sept 11," "terrorist attacks." | 1979, 1981, 1985, 1986, 1987, 1988, 1989, 1990, 1991, 1992, 1993, 1994, 1995, 1996, 1997, 1998, 1999, 2000, 2001, 2002, 2003, 2004, 2005, 2006, 2007, 2008, 2009, 2010, 2011, 2012, 2013, 2014, 2015, 2016, 2017, 2018, 2019, 2020, 2021, 2022, 2023, 2024 |
| Response and Preparation | (n = 13) "anthrax vaccine," "biodefense," "chemical suits," "gas masks," "immunizations," "inoculated," "medical research," "military response," "preparation," "protection biological weapons," "public health," "response," "vaccination." | 1985, 1986, 1987, 1989, 1990, 1991, 1992, 1993, 1994, 1995, 1996, 1997, 1998, 1999, 2000, 2001, 2002, 2003, 2004, 2005, 2006, 2007, 2008, 2009, 2010, 2011, 2012, 2013, 2014, 2015, 2016, 2017, 2018, 2019, 2020, 2021, 2022, 2023, 2024 |
